# Efficacy and Mode of Action of Mesenchymal Stem Cells in Non-Ischemic Dilated Cardiomyopathy: A Systematic Review

**DOI:** 10.3390/biomedicines8120570

**Published:** 2020-12-05

**Authors:** Cecilie Hoeeg, Sabina Frljak, Abbas Ali Qayyum, Bojan Vrtovec, Jens Kastrup, Annette Ekblond, Bjarke Follin

**Affiliations:** 1 Cardiology Stem Cell Centre, Department of Cardiology, Centre for Cardiac, Vascular, Pulmonary and Infectious Diseases, Rigshospitalet, University of Copenhagen, Henrik Harpestrengs Vej 4, 2100 Copenhagen, Denmark; abbas.ali.qayyum@regionh.dk (A.A.Q.); jens.kastrup@regionh.dk (J.K.); annette.ekblond@regionh.dk (A.E.); bjarke.follin@regionh.dk (B.F.); 2 Advanced Heart Failure and Transplantation Center, University Medical Center Ljubljana, Zaloska 7, 1000 Ljubljana, Slovenia; sabina.frljak@gmail.com (S.F.); bojan.vrtovec@gmail.com (B.V.); 3 Department of Immunology and Microbiology, Faculty of Health and Medical Sciences, University of Copenhagen, Blegdamsvej 3B, 2200 Copenhagen, Denmark

**Keywords:** dilated cardiomyopathy, mesenchymal stem cells, mode of action, regeneration

## Abstract

Non-ischemic dilated cardiomyopathy (NIDCM) constitutes one of the most common causes to non-ischemic heart failure. Despite treatment, the disease often progresses, causing severe morbidity and mortality, making novel treatment strategies necessary. Due to the regenerative actions of mesenchymal stem cells (MSCs), they have been proposed as a treatment for NIDCM. This systematic review aims to evaluate efficacy and mode of action (MoA) of MSC-based therapies in NIDCM. A systematic literature search was conducted in Medline (Pubmed) and Embase. A total of 27 studies were included (3 clinical trials and 24 preclinical studies). MSCs from different tissues and routes of delivery were reported, with bone marrow-derived MSCs and direct intramyocardial injections being the most frequent. All included clinical trials and 22 preclinical trials reported an improvement in cardiac function following MSC treatment. Furthermore, preclinical studies demonstrated alterations in tissue structure, gene, and protein expression patterns, primarily related to fibrosis and angiogenesis. Consequently, MSC treatment can improve cardiac function in NIDCM patients. The MoA underlying this effect involves anti-fibrosis, angiogenesis, immunomodulation, and anti-apoptosis, though these processes seem to be interdependent. These encouraging results calls for larger confirmatory clinical studies, as well as preclinical studies utilizing unbiased investigation of the potential MoA.

## 1. Introduction

Non-ischemic dilated cardiomyopathy (NIDCM) is a disease affecting the myocardial tissue and represents one of the most common causes to non-ischemic heart failure [1]. It is characterized by systolic dysfunction and left ventricle (LV) or biventricular dilatation, in the absence of factors normally involved in global systolic impairment, including coronary artery disease, hypertension and valve disease [2]. The prevalence of NIDCM remains uncertain, though estimates suggest the number to be between 1:250 and 1:2500 [3,4].

NIDCM has a diverse aetiology including genetic disorders, exposure to drugs and toxins, infectious agents, peripartum, and autoimmune disorders. Additionally, an idiopathic variant of NIDCM exists [2,5]. One group of drugs known to cause NIDCM is anthracyclines, which are used to treat several cancers [6,7]. During the first 10 years after treatment, severe cardiotoxicity occurs in approximately 6% of patients and subclinical cardiotoxicity in 18% [8]. Consequently, the growing population of cancer survivors will leave an increasing number of patients at risk of developing NIDCM. Despite the diverse aetiology, there seems to be common tissue denominators, including interstitial fibrosis, immune cell infiltration, microvascular injury, and degenerated cardiomyocytes [2,9,10,11]. As the disease progresses over time, the patients are left at risk for developing non-ischemic heart failure [12]. Despite optimal medical therapy, the condition causes severe morbidity and mortality, due to a continuous decline in cardiac pump function, resulting in fatigue, dyspnea, reduced working capacity, and poor quality of life [10,12,13,14]. This makes NIDCM the most common indication for heart transplantation [10,13,14]. Thus, there are unmet needs for additional treatment strategies to improve mortality and quality of life for patients.

A promising new treatment strategy is cell therapy using mesenchymal stem cells (MSCs). MSC therapy has been extensively investigated in preclinical and clinical studies with ischemic heart disease, providing evidence that MSCs possess anti-fibrotic, angiogenic, and trophic effects [15,16,17,18,19,20]. During the past decade, these properties have led researchers to investigate MSC therapy for NIDCM, due to the fibrotic, degenerative, and immunological components of the disease [10,21].

At this point, most data available is from animal studies, using experimentally induced NIDCM, and a few clinical trials. The majority of these studies report improvement in cardiac function, including left ventricular ejection fraction (LVEF) and LV end diastolic diameter and volume [22,23]. Despite conduction of these studies, the scientific rationale for using MSC therapy to treat NIDCM, including comprehensive knowledge on efficacy and regenerative mechanisms, has still to be clarified.

The present systematic review aims to evaluate the efficacy and mode of action (MoA) of MSC therapy in NIDCM. Using published clinical trials and animal studies, this review highlights important cellular and molecular mechanisms involved in regeneration, as well as point towards areas in need of further investigation.

## 2. Experimental Section

### 2.1. Study Protocol, Search Strategy, and Data Sources

**Figure 1:**
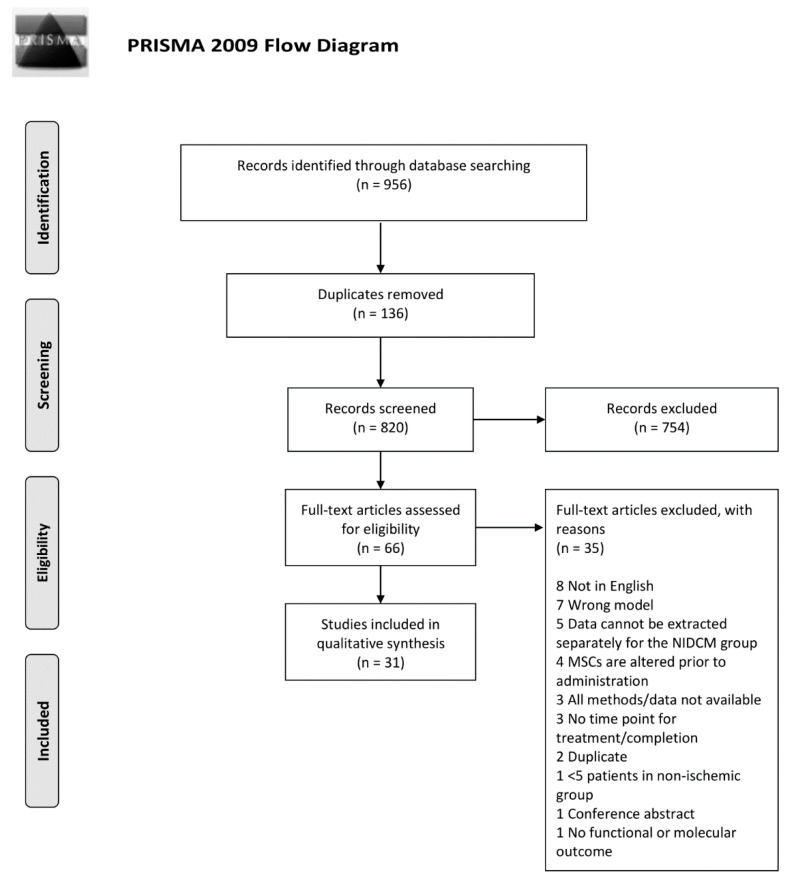
PRISMA flow chart illustrating the inclusion process of literature. PubMed and Embase were used for identification of existing literature.

The objectives of the literature search were specified using the PICO criteria, including details on population, intervention, comparator, and outcome (Appendix A). The complete study protocol was subsequently developed and contained detailed criteria for study selection, eligibility, and data extraction. The review was conducted in accordance with the PRISMA guidelines (Preferred Reporting Items for Systematic Reviews and Meta-Analysis) (Figure 1) (Moher et al., 2009). The PRISMA checklist can be found in Appendix B.

Two electronic databases were searched; Medline (PubMed) and Embase. The last search in both databases, was performed on 20 February 2020. The literature search was restricted to articles published in English. In PubMed, the applied MeSH terms were Mesenchymal Stem Cells AND Cardiomyopathy, Dilated OR Heart Failure, and all entry terms were included as free text. For the search in Embase, the following medical subject headings were included: Mesenchymal stem cell AND Congestive cardiomyopathy OR Heart failure. All narrower terms were included using the explode function. Both search syntaxes are provided in Appendix C.

Using Covidence online software, two independent reviewers screened all titles/abstracts, retrieved from the initial search, and subsequently all full texts (CH, BF). Discrepancies regarding inclusion were resolved by a third reviewer (SF).

### 2.2. Study Criteria

Published clinical and animal studies analyzing the use of MSC therapy in NIDCM were included. For animal studies, both medically induced NIDCM, genetic and inflammatory NIDCM models were included to represent the heterogeneity of the disease.

The predefined exclusion criteria for title/abstract screening were: (1) irrelevant to the subject of the study, (2) reviews and meta-analysis, and (3) letters to the editors and editorials. For full text screening they included: (a) wrong animal model or a suitable model but treatment prior to the onset of NIDCM phenotype, (b) data could not be extracted separately for NIDCM patients, (c) MSCs were differentiated or altered genetically for enhancement purposes, (d) no information regarding time point for treatment/completion was reported, and (e) full text/data not available. Studies administering labelled cells were included, but data was initially extracted and analyzed separately to accommodate the risk of labelling affecting cell function.

### 2.3. Data Extraction

To obtain the most comprehensive knowledge on MoA, different study types and outcomes were included, emphasizing measures for cardiac function and tissue, cellular, and molecular responses to treatment. Due to the heterogeneity in reporting of outcomes among eligible studies, meta-analysis was not attempted. Thus, the present systematic review aims to give a descriptive presentation of available data.

### 2.4. Protein-Protein Interaction Network

To elucidate key factors in MSC MoA, proteins significantly altered by treatment were subjected to Search Tool for the Retrieval of Interacting Genes/Proteins (STRING) v11, applying a minimum required interaction score of 0.400 [24]. Data on protein expression from both clinical and preclinical studies were pooled, based on the assumption that the biological function of specific proteins are the same between species. The database comprises interaction records from curated websites, including Reactome, BioCyc, KEGG, and Gene Ontology as well as legacy data from PID and BioCarta [24]. The analysis shows known and predicted protein–protein interactions (PPI), in which the proteins are denoted by nodes and interactions by edges. To further explore the PPIs, biological processes associated with the protein expression were color coded.

### 2.5. Assessment of Study Quality and Publication Bias

Due to the inclusion of both clinical and animal studies, a modified SYRCLE’s risk of bias (RoB) tool was applied [25]. Random sequence generation, baseline characteristics (animal studies), allocation concealment, blinding of participants and personnel, blinded outcome assessment, incomplete outcome data, and other bias were included in the tool. All eligible studies were assessed on all parameters and rated with either high RoB, low RoB, or not reported. Two independent reviewers rated all studies (CH and BF) and discrepancies regarding RoB assessment were resolved by a third reviewer (AQ).

## 3. Results

### 3.1. Study Characteristics and Quality Assessment

The literature search initially yielded 956 studies, from which 134 were duplicates and thus removed. First, titles and abstracts were screened for relevancy and study type. Patient populations with ischemic heart disease or animal models of acute myocardial infarction were the main reasons for exclusion, leaving 66 studies for full-text evaluation. Ultimately, a total of 31 studies met the predefined inclusion criteria. Seven studies reporting data on the use of MSCs in clinical trials with NIDCM were included, though data from five of the studies originated from the same trial [12,26,27,28,29]. To ensure that original data was only reported and analyzed once, publications from the same trial was considered as one study for the remaining analysis, giving a total number of 27 included studies.

The number of participants in the clinical trials ranged from 27 to 53 patients and follow-up ranged from one-week [30], to 12 months [12,23] post treatment. A total of 24 preclinical studies were included, with rodent models being the primary species used for NIDCM induction (Appendix D). Three studies used an autoimmune phenotype [31,32,33] and two a genetic phenotype [34,35]. The remaining studies applied a medically induced NIDCM phenotype.

### 3.2. Risk of Bias

A quality assessment was performed on all included studies and is presented in Appendix E. Regarding the preclinical studies, most studies report baseline characteristics, thus minimizing RoB in functional data. However, preclinical studies tend to omit information on random sequence generation and allocation concealment. Additionally, limited information was available on blinding of outcome assessment.

Five of the included animal studies had generally high RoB, as they scored low RoB in ≤1 of the assessed parameters [36,37,38,39,40]. Despite this, they reported the same treatment effect as the remaining studies. As study design generally tends to be insufficiently described in animal studies, none of them were excluded. If an outcome, however, was only reported in a high RoB study, it is noted in the text.

For the three included clinical trials, more information was available on study design, including random sequence generation and blinded outcome assessment, thus reducing RoB. Information on allocation concealment is here too sparse, which can be explained by some of the studies being open-labelled.

### 3.3. Cell and Transplant Type

A total of 21 of the included trials tested bone marrow derived MSCs (BM-MSCs), four tested human umbilical cord blood MSCs (hUCB-MSCs) and three tested adipose tissue–derived mesenchymal stem cells (AT-MSCs) (Appendix D). Two studies compared AT-MSCs and BM-MSCs [32,41]. Due to the frequency of BM-MSCs across studies, the results presented in this review primarily reflects the properties of this cell type. Despite this, both BM-MSCs, AT-MSCs, and hUCB-MSCs improved cardiac function and initiated anti-fibrotic, angiogenic, and immunomodulatory mechanisms [32,35,41]. Additionally, BM-MSCs and hUCB-MSC displayed anti-apoptotic properties [35]. As all MSC subtypes exhibited comparable features, it is reasonable to suggest that MoA is the same independent of cell origin.

Another aspect to consider is transplant type. A total of fourteen studies used syngeneic transplants, six used xenogeneic transplants, four used autologous transplants and three used allogeneic transplants. Recently, allogeneic MSCs have gained focus as a potential off-the-shelf therapy as they are immune evasive [12,20]. The POSEIDON-DCM trial compared autologous and allogeneic BM-MSCs and found allogeneic transplants to be superior in increasing LVEF and decreasing tumor necrosis factor α (TNF-α) [12]. Furthermore, autologous MSCs may have a reduced regenerative capacity, due to the underlying aetiology of the patient. This obstacle is avoided with allogeneic transplants from healthy donors. Despite this, most animal studies have used syngeneic and autologous transplants, while the three clinical trials have applied autologous and allogeneic transplants.

### 3.4. Administration Route

Different administration routes have been applied in the included studies. 14 studies used intramyocardial (IM) injection, 10 intravenous (IV) injection, two used intracoronary (IC) injection and one used injection in the hind limb muscle. Studies using IM and IV injections were equally distributed across outcomes related to cardiac function, fibrosis, angiogenesis, immunomodulation, and apoptosis. However, only one study using IV injections showed that MSC therapy led to altered fibrotic gene expression [22]. In spite of this, IV injections led to reduced cardiac fibrosis in five studies, suggesting that molecular alterations were present.

Overall, the included studies do not reflect any significant changes between IM and IV injections. There is, however, clinical evidence of improved retention and functional outcome in NIDCM patients with IM delivery compared to IC administration of CD34+ cells [42]. This might be similar for MSCs. However, direct meta-analysis and comparison of delivery routes was not within the scope of this review.

### 3.5. Cell Labelling

In order to track MSCs in vivo, cell labelling was applied in 13 out of the 27 included studies. Only one study stated that labelling did not affect cell viability and function [31,35,38,39,40,41,43,44,45,46,47,48,49]. As the effect of labelling was not addressed in 12 out of 13 studies, the initiating analysis was performed on labelled and unlabeled cells separately. Prior to the final synthesis of results, we evaluated outcomes in all studies using labelled cells, and compared data to studies using unlabeled cells. All included studies, except two, reported improved cardiac function, suggesting that labelling did not affect the overall MSC function [45,50]. Approximately half of the studies reporting anti-fibrotic and angiogenic properties of MSCs used labelled cells, indicating that these properties remained intact [31,35,38,39,41,43,44]. Two out of three studies investigating apoptosis, reference [35,49] and two out of three investigating immunomodulation [35,41] used labelled cells and reported similar tendencies. Consequently, there was no evidence of labelling affecting cell function, thus data was assessed coherently for the remaining analysis.

### 3.6. Effect on MSC Therapy on Cardiac Function

#### 3.6.1. Clinical Evidence of Functional Effect

Fatkhudinov et al. [30] evaluated the effect of allogeneic MCSs in 27 patients with NIDCM, advanced heart failure, and LVEF < 35%. A total of 14 patients were treated conservatively, and 13 underwent surgical procedure. Both groups were subdivided into a group receiving IC cell transplantation and a control group. All patients were followed for 12 months. MSC transplantation was associated with improved 6-min walk test and NYHA class, reaching maximum effect by month three (numerical values not provided, *p* < 0.05). Increased LVEF was noted in the MSC group but did not reach statistical significance. No change in left ventricular size or volume was present.

Xiao, et al. [23] compared the efficacy of IC administration of bone marrow mononuclear cells (BM-MNCs) or BM-MSCs in patients with NIDCM and LVEF < 40%. A total of 53 patients were randomized into three groups receiving IC infusion of BM-MNCs (*n* = 16), BM-MSCs (*n* = 17) or normal saline (*n* = 20). Patients in the BM-MSC group exhibited a significant improvement in cardiac function, as LVEF increased from 34.1 ± 3.6 to 41.4 ± 5.1 at three-month follow-up (*p* < 0.05) and to 41.0 ± 6.7 at 12-month follow-up (*p* < 0.05). Furthermore, NYHA class decreased from 2.7 ± 0.7 to 1.7 ± 0.7 and 1.9 ± 1.1 at three- and 12-month follow-up, respectively (*p* < 0.05). Patients receiving BM-MNCs also presented with improvement in LVEF and NYHA class, but less prominent and only statistically significant at three-month follow-up.

Hare et al. [12] performed the first randomized trial directly comparing the effects of autologous and allogeneic BM-MSCs therapy in NIDCM (POSEIDON-DCM: Percutaneous Stem Cell Injection Delivery Effects on Neomyogenesis in Dilated Cardiomyopathy). A total of 37 patients with stable heart failure and LVEF < 40% were randomized in a 1:1 ratio to receive transendocardial injections of a fixed dose (100 × 10^6^) of either autologous or allogeneic MSCs. After 12 months, LVEF of patients receiving allogeneic MSCs had significantly improved by 8.0 percentage points (*p* = 0.004) and the 6-min walk-test had improved by 37 m (*p* = 0.04). Patients receiving autologous MSCs did not improve to the same extent, as LVEF increased 5.4 percentage points (*p* = 0.116). Furthermore, the 12-months all-cause rehospitalization rates and the rate of major adverse cardiovascular events were significantly lower with allogeneic MSC therapy (28.2% and 20.3%, respectively) (*p* < 0.05) compared to autologous (70% and 57.1%, respectively). A sub-analysis by Florea et al. [29] demonstrated that the effects of MSC therapy on cardiac function and clinical outcomes are comparable in male and female patients. This finding was present despite differences in baseline clinical characteristics. In addition, genetic sequence analysis revealed that the effect of MSC treatment was associated with genetic variants, including mutations in the cytoskeleton, nuclear membrane, sarcomere, and mitochondria. At 12-month follow-up, LVEF increased by 13.6% in the patients with no pathological variants (*n* = 6, *p* = 0.002), compared to variants of uncertain significance (+6.5%, *n* = 20, *p* = 0.005), and patients positive for pathological variants (−5.9%, *n* = 8, *p* = 0.2).

This suggests that the genetic profile of NIDCM patients plays a role in responsiveness to MSC therapy, and that genetic testing can be used before considering this therapy [26].

#### 3.6.2. Preclinical Evidence of Functional Effect

**Figure 2:**
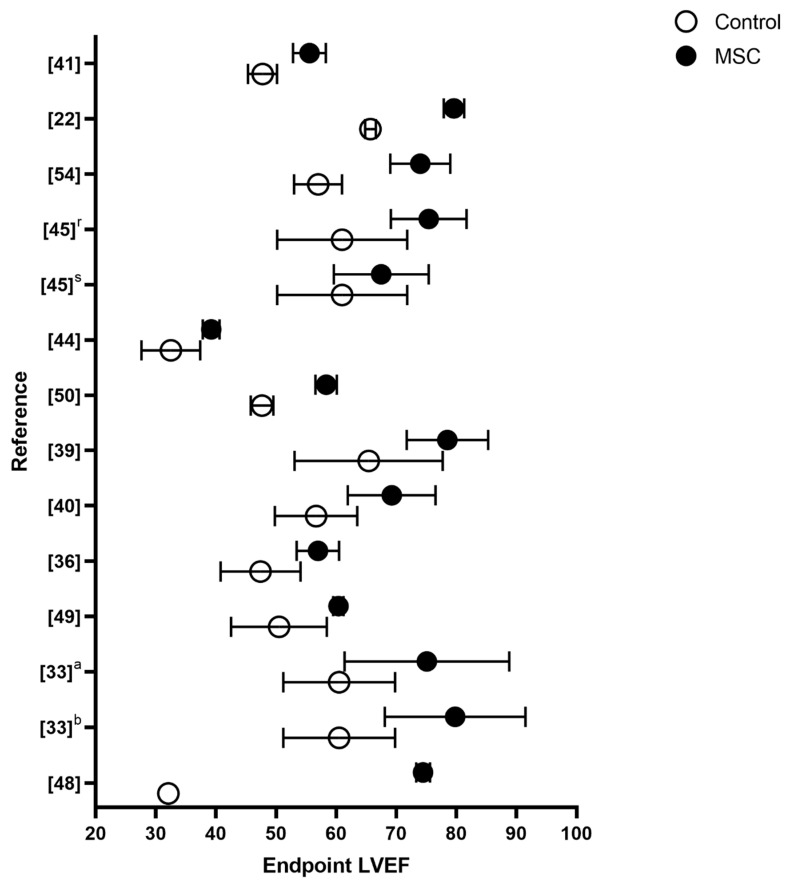
Overview of LVEF values for each preclinical study providing numerical endpoint LVEF values. The preclinical studies not reporting on this are not included in the figure. The y-axis is presented with reference number. Additionally, r is repeated injections, s single injection, a AT-MSCs and b BM-MSCs. Data from both MSC and control croup is presented as LVEF mean with standard deviations.

One preclinical study did not investigate functional outcomes [33], and two studies found no functional effect of the MSC treatment [45,50]. The remaining 21 included preclinical studies reported a significant effect on functional parameters following MSC treatment, primarily measured as LVEF. Figure 2 illustrates the preclinical studies reporting numerical values for LVEF at follow-up. Most studies reporting both mean and standard deviation found the difference in LVEF to be between 15 and 6 percentage points, when comparing MSC groups to controls at follow-up [22,35,48,49]. Studies including baseline values reported ΔLVEF between 25.2 and 1.9 for the treatment groups, depending on the animal model, with a median at 13.6. It was not within the scope of this review to perform a meta-analysis. However, a recent meta-analysis by Lopes et al. [51] found MSC therapy to result in a weighted difference of 10.4 (7.24–12.84) percentage points in LVEF compared to controls. This is in accordance with the clinical results from POSEIDON-DCM on ΔLVEF, with allogeneic treatment of patients without pathological variants. Ventricular pressure was measured in six studies [31,33,36,41,46,48]. In five of these studies, MSC treatment resulted in significantly increased dP/dt, indicating increased cardiac contractility. This finding is consistent with the observed improvements in LVEF. Additionally, LV end diastolic pressure was significantly decreased in the three studies reporting on this outcome [31,33,36]. Arterial blood pressure was increased to normal levels in Ammar et al. [41] and tended to normalize in Psalitis et al. [43].

Taken together, the evidence suggests that MSC treatment improves functional outcomes of cardiac pump function and blood pressure. This is solid evidence for initiating phase I clinical trials. However, knowledge about MoA is necessary to move into larger clinical studies. With this in mind, we investigated the published evidence on MoA.

### 3.7. Effect on MSC Therapy on Cardiac Regeneration

**Figure 3:**
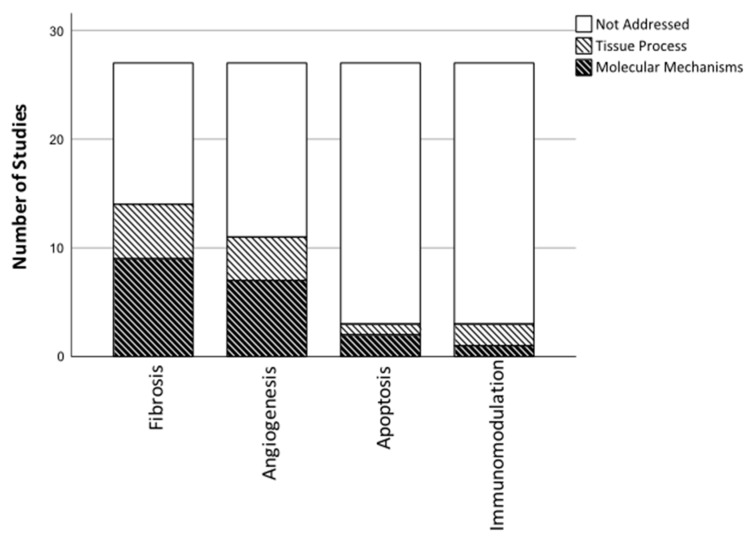
Illustrates the number of studies that have investigated specific regenerative tissue processes, and potential underlying molecular mechanisms.

Despite the distinct outcomes included in the present review, the majority was related to four aspects of cardiac regeneration including, fibrosis, immunomodulation, angiogenesis, and apoptosis (Figure 3).

#### 3.7.1. Fibrosis

Data extraction revealed that cardiac fibrosis and the anti-fibrotic effects of MSCs have received great attention. A total of 14 out of 27 studies evaluated cardiac fibrosis using immunohistochemistry (IHC) and analyzed gene and protein expression patterns related to this process (Figure 3).

Results demonstrated that MSC therapy alters the fibrotic process in NIDCM on both a tissue and molecular level [32,43,44]. MSC treatment significantly attenuated myocardial fibrosis, by reducing collagen volume fraction (CVF) and improving myocardial fiber alignment on IHC. This is, together with the positive effect on cardiac function, the most consistent finding, reported by all but one study investigating fibrosis [22,31,32,34,35,36,37,39,43,44,47,49,52].

**Figure 4:**
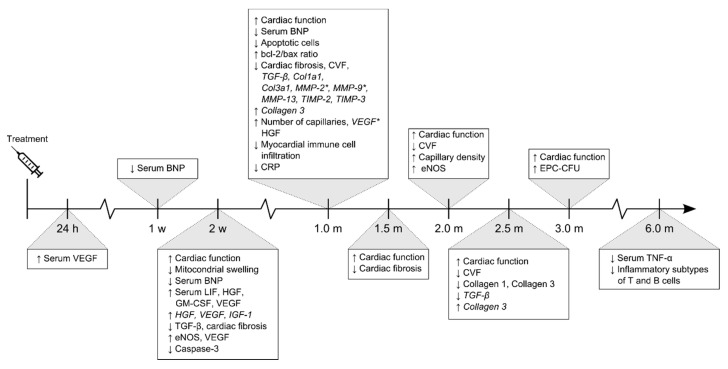
Timeline illustrating functional and molecular outcomes investigated in the included studies. Five studies investigated molecular outcomes within the first four weeks after treatment, whereas the remaining investigated at one-month post treatment or later. Gene expression in italics, asterisk (*) depicts dual protein and gene expression, ↑ represents and upregulation and ↓ a downregulation. Abbreviations; Brain natriuretic peptide (BNP), Leukocyte inhibitory factor (LIF), Granulocyte- macrophage colony-stimulating factor (GM-CSF), Insulin-like growth factor-1 (IGF-1), Collagen Type III Alpha 1 Chain (Col3a1), C-reactive protein (CRP).

Looking at gene and protein expression patterns, MSC transplantations significantly downregulated the gene expression of collagen 1 and 3 and transforming growth factor β (TGF-β) in the cardiac tissue four weeks after treatment (Figure 4) [34,35,38,39,52]. At 10 weeks, the gene expression of collagen 3 was upregulated, but the protein expression reduced, which might be explained by a temporal shift [22]. These findings indicate that MSC transplantations inhibit collagen transcription and subsequently collagen synthesis and deposition, resulting in the reduced CVF. In addition to this, Deng et al. [52] reported that MSC transplantation reduced TGF-β transcription with 88.8% (*p* < 0.05). This finding was supported by Yu et al. [53], which likewise found inhibited TGF-β transcription following MSC treatment. An increased TGF-β expression is often associated with activation of fibrotic pathways; hence, the attenuated cardiac fibrosis may partially be mediated by alterations of TGF-β signaling [49,54].

Another aspect of ventricular remodeling is turnover of fibrotic tissue, which is partially regulated by matrix metalloproteinases (MMPs) and tissue inhibitors of metalloproteinases (TIMPs) [38]. Several studies found that MSC treatment significantly reduced the gene and protein expression of MMP-2 and MMP-9 [31,34,38,39]. However, MMP-9 reduction was only reported in a study scoring high RoB [38,39]. Shabbir et al. [34] found reduced mRNA expression of MMP-9 (*p* < 0.001), MMP-13 (*p* < 0.01), TIMP-2 (*p* < 0.05) and TIMP-3 (*p* < 0.05), compared to the NIDCM control, thus reversing the pathological expression profile associated with NIDCM. In the failing heart, both MMPs and TIMPs contribute to adverse remodeling by degrading normal collagens, which is subsequently replaced by interstitial fibrosis comprising poorly cross-linked collagens [39]. By downregulating the expression of MMPs and TIMPs, MSCs may inhibit the progression of ventricular remodeling and dilation, thus improving cardiac function. Together these findings substantiate the anti-fibrotic properties of MSCs and the advantage of applying them therapeutically to target the fibrotic nature of NIDCM.

#### 3.7.2. Immunomodulation

Only three out of the twenty-seven included studies analyzed outcomes related to the immune system; two preclinical studies and POSEIDON-DCM (Figure 3) [12,35,41].

Using a doxorubicin induced NIDCM phenotype in diabetic rats, Ammar et al. [41] found that MSC transplantations significantly reduced % area of immune cell infiltration in the myocardium (*p* < 0.05). Using a genetic phenotype, Gong et al. [35] established that MSCs significantly reduced serum C-reactive protein (*p* < 0.05). The POSEIDON-DCM study found that treatment with allogeneic MSCs significantly decreased serum levels of TNF-α with −10.6 ± 1.6 pg/mL at six-months follow-up (*p* < 0.0001). Elevated serum levels of TNF-α are associated with progression of heart diseases, therefore, by reducing pro-inflammatory cytokines in the myocardium, MSCs may shift the microenvironment towards an anti-inflammatory profile [12]. The study likewise found that allogeneic MSC therapy altered the humoral lymphocyte profile by reducing subtypes of both B and T cells, normally associated with chronic inflammation. Considering the fundamental role of the immune system in NIDCM and the immunomodulatory properties of MSCs, surprisingly few studies have reported on this aspect.

#### 3.7.3. Angiogenesis

From the included studies, 11 out of 27 have analyzed outcomes related to angiogenesis. Microscopically, studies have demonstrated that MSC transplantations increased number and density of vessels in the myocardium [31,32,34,35,41,43]. These findings suggest that MSC transplantations activate an angiogenic response, leading to increased myocardial neovessel formation. On a molecular level, MSCs increased the cardiac gene expression of vascular endothelial growth factor (VEGF), which translated into increased serum VEGF [32,34,35,39,44,55] VEGF is an important signaling protein secreted to stimulate neovessel formation, thus the increased vessel density may be partly due to the increased VEGF production [32]. Additionally, MSC treatment has been shown to increase the circulating levels of hepatocyte growth factor (HGF), a potent angiogenic factor [31,34,55]. This finding was also present on a transcriptional level, likely mediating the increased circulating HGF [39]. This finding was, however, only reported in a study scoring high RoB.

Another aspect of the angiogenic response is endothelial function [12]. Endothelial dysfunction is a significant feature of heart failure, leading to diminished endothelial progenitor cell function and flow-mediated vasodilation (FMD) [27]. The POSEIDON-DCM trial demonstrated that allogeneic MSC therapy significantly improved endothelial function by increasing endothelial progenitor colony forming units (*p* = 0.0107) and FMD% (*p* = 0005) at three months compared to baseline [12]. Studies revealed that MSC therapy increased the ventricular protein expression of endothelial nitric oxide synthase (eNOS) (*p* < 0.05), an enzyme important for proper endothelial function [47,49]. Furthermore, eNOS was significantly decreased in the NIDCM control group, which is associated with reduced myocardial neovascularization and impaired endothelium-dependent vasodilation, thus supporting the results from POSEIDON-DCM [49]. The ability of allogeneic MSCs to restore endothelial function, together with the alterations in angiogenic factors, offer new insights into MSC-induced angiogenesis.

In all, these findings provide solid evidence that MSC therapy induces angiogenesis in NIDCM, likely stimulated by an increased paracrine secretion and improved endothelial function.

#### 3.7.4. Apoptosis

Three out of the included twenty-seven studies investigated outcomes related to cell survival and apoptosis [34,35,49]. Shabbir et al. [34] performed IHC on myocardial tissue sections, which showed that MSC therapy reduced apoptotic cardiomyocytes in NIDCM hearts by approximately 60% (*p* < 0.01) compared to controls. On a molecular level, MSC therapy increased the ventricular B-cell lymphoma 2 (Bcl-2)/Bcl-2-associated X protein (Bax) ratio (*p* < 0.01) and reduced protein expression of Caspase-3 compared to NIDCM controls (*p* < 0.05) [35,49]. Bcl-2 is an important inhibitor of apoptosis among ventricular cardiomyocytes, whereas Bax is a pro-apoptotic protein. Consequently, an increased Bcl-2/Bax ratio suggests inhibition of pathways involved in cardiac apoptosis [35]. Caspase-3 is activated during cell apoptosis and has specifically been associated with doxorubicin administration in vivo [56]. However, Mohamed et al. [49], which reported reduced Caspase-3 protein levels following MSC treatment, used an Isoproterenol-induced NIDCM phenotype. These findings suggest that the same mechanisms are active in both NIDCM models, and beyond this, that MSC-mediated inhibition of Caspase-3 may reduce cardiomyocyte apoptosis and subsequently improve cardiac function.

#### 3.7.5. MSC Mode of Action in NIDCM

The results demonstrate that most of the included studies have evaluated MSC efficacy and MoA approximately one month following treatment (Figure 4 and Appendix D). At this timepoint studies found improved cardiac function, reduced fibrosis, and increased myocardial capillary density. These tendencies suggest that the molecular and cellular mechanisms underlying these effects, have been initiated within the first weeks after treatment.

When reporting on MoA, studies most commonly discuss specific regenerative mechanisms as isolated processes. However, when analyzing outcomes associated with fibrosis, angiogenesis, apoptosis, and immunomodulation, it becomes evident that these processes are mutually connected. To exemplify this, the POSEIDON-DCM trial found that allogeneic MSCs reduced serum TNF-α, while Mohamed et al. [49] reported reduced ventricular Caspase-3 protein following treatment [12]. Interestingly, release of TNF-α, has been described to activate Caspase-3 and subsequently stimulate progression of cardiomyocyte apoptosis. It is therefore likely that the reduced Caspase-3 is in part mediated by a decreased serum TNF-α, thus shifting the inflammatory microenvironment and alleviating cardiomyocyte apoptosis [12,35,49]. The biological properties of TGF-β likewise exemplifies the complexity of MSC-mediated cardiac regeneration. Most of the included studies describe its involvement in cardiac fibrosis, thus suggesting that downregulation is beneficial [10,52,53]. However, TGF-β is also described to be anti-inflammatory, as it can promote differentiation of anti-inflammatory macrophages and inhibit cytotoxic T cells in the damaged heart [20,27,28]. Based on this, reporting increased or decreased TGF-β expression to be solely beneficial or detrimental may be oversimplified and not reflective of the complex processes in vivo.

**Figure 5:**
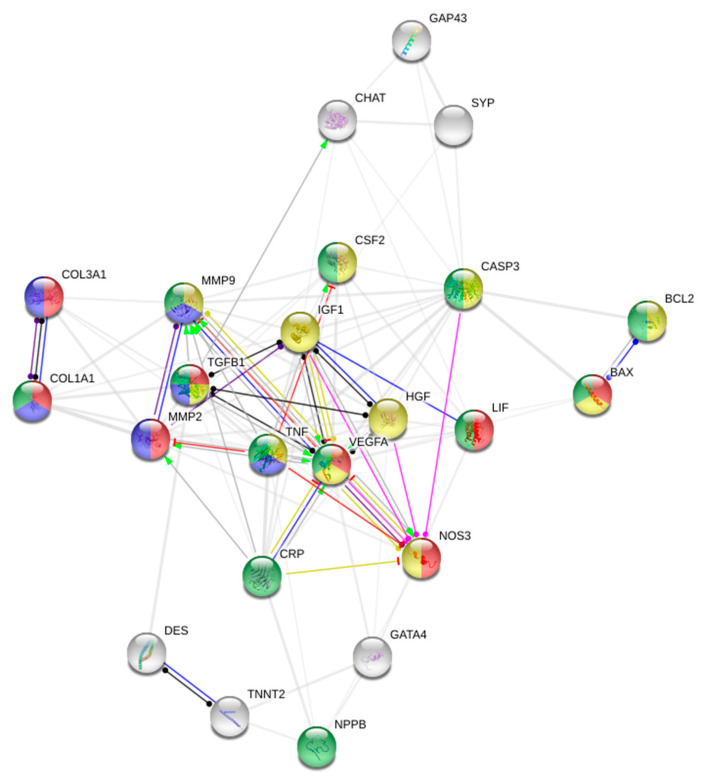
The PPI network. Red are proteins involved in blood vessel development, purple ECM organization, green regulation of immune system processes and yellow apoptosis. Red interaction lines illustrate inhibition, green activation, pink post-translational modification, blue binding, purple catalysis, yellow transcriptional regulation, and black reaction. Abbreviations; Transcription factor GATA-4 (GATA4), Brain natriuretic peptide (NPPB), Growth Associated Protein 43 (GAP43), Cholineacetyltransferase (CHAT), Synaptophysin (SYN), Leukocyte inhibitory factor (LIF), Granulocyte-macrophage colony-stimulating factor (CSF2), Insulin-like growth factor-1 (IGF-1), Troponin T (TNNT2), Collagen Type III Alpha 1 Chain (Col3a1), Collagen Type 1 Alpha 1 Chain (Col1a1), Endothelial NOS (NOS3), C-reactive protein (CRP), Caspase 3 (CASP3), Desmin (Des).

To gain further knowledge on the complex biological processes initiated by MSC therapy, a STRING analysis was performed. The analysis provided a PPI enrichment *p*-value of < 1.0 × 10^−16^, indicating that the proteins, whose expression was altered by treatment, are biologically connected, and not randomly occurring. The connectivity also points towards that similar processes are initiated following MSC treatment, despite varying MSC types and NIDCM models. As illustrated in Figure 5, several of the proteins are implicated in numerous physiological processes including ECM organization (purple nodes) and angiogenesis (red nodes). These mechanisms are most likely accountable for the observed increase in vessel density and decrease in cardiac fibrosis. The central placement and multiple connections of VEGFA, TNF-α, and IGF-1 point towards the initiated mechanisms being conducted through regulation of these factors. However, knowledge regarding which cell populations are responsible for the changed proteins levels is poorly investigated. Due to the notoriously low retention rates of MSCs in the heart, it is likely that the examined proteins are secreted by endogenous cell populations and not MSCs themselves [42].

Though little has been reported on immunological and apoptotic markers, the STRING analysis support that MSC therapy exerts immunomodulation (green nodes) and alters apoptotic processes (yellow nodes) in NIDCM. However, the downstream effects of these remain uncertain, underpinning the need for deeper exploration of MoA. All things considered, the effect of MSC therapy cannot be ascribed to one single growth factor or limited to one physiological process, but instead is the result of different regenerative processes, which may act synergistically [57].

Despite fibrosis, angiogenesis, apoptosis, and immunomodulation being the primary focus of existing studies, other aspects of MSCs may be fundamental to the observed improvement in cardiac function. Oxidative stress has been described to be one of the major mechanisms through which the anthracycline, doxorubicin, injures the heart [10,56]. Doxorubicin interacts with eNOS, and with increasing concentrations, eNOS can switch from generation of nitric oxide (NO) to superoxide, a reactive oxygen species contributing to endothelial dysfunction [28,47,56]. Endothelial function is often measured using endothelial progenitor cell-colony forming units (EPC-CFU), and has been found to be inversely correlated with serum TNF-α. Administration of MSCs increased peripheral blood EPC-CFUs, reduced serum TNF-α, and normalized ventricular eNOS protein expression. These findings suggest that MSC therapy can alleviate anthracycline-induced endothelial dysfunction and oxidative stress, possibly by restoring eNOS function [12,28,47,49]. Thus, the functional improvement may partially be caused by improved endothelial function and reduced oxidative stress. Using the STRING analysis, it emerged that seven of the included proteins were implicated in oxidative stress and ROS regulation, strengthening this hypothesis.

## 4. Challenges, Limitations and Future Perspectives

At this point, only a few small clinical trials have been conducted. Though the results are encouraging, there is a need for larger, international, trials, enabling inclusion of more patients. These should be performed to confirm the beneficial effects of MSC treatment in NIDCM patients and move forward in the drug development pipeline. However, to initiate larger trials, more knowledge on MoA is required.

As stated previously, 12 out of 13 studies did not analyze the effect of cell labeling on MSC viability and function. Since current literature reports labelling to affect these exact two properties, this issue should be evaluated in future studies attempting to address MoA [58,59]. If the fundamental functions are in fact altered, the results presented here may not uncover the full potential of MSC therapy in NIDCM.

The included studies show that MSC therapy improves cardiac function, ameliorates myocardial fibrosis and stimulates angiogenesis [31,32,34,35,41,43]. Despite the solid evidence of these properties, most studies build upon histochemical evaluation of cardiac tissue sections taken from animals, in which the same outcomes are addressed at approximately the same timepoint (Figure 3 and Figure 4). This tendency elucidates the reproducibility of the results but fails to provide further mechanistic insight. Additionally, little attention has been paid towards the immunomodulation. This aspect of MSC-mediated regeneration in NIDCM, thus, seems an evident topic for future research on MoA, due to the fundamental role of the immune system in NIDCM. However, as the immunomodulatory properties are extensively described in other cardiac diseases, including ischemic heart disease, one may raise the question, if results are excluded from published articles due to non-significant findings or simply lack of focus on this topic [17,20,60]. All things considered, the existing MoA data favors a more explorative approach in future research, in which the immediate molecular and especially cellular processes should be prioritized. Furthermore, it is fundamental that the currently known MoA is being evaluated in future clinical trials, in order to translate findings into human.

## 5. Conclusions

MSC therapy has emerged as a promising treatment strategy for patients with NIDCM, due the degenerative nature of the disease and the regenerative properties of MSCs. The present systematic review provides evidence that MSC therapy has the potential to improve cardiac function, reduce myocardial fibrosis and increase angiogenesis. Further insight into MoA displays that MSCs induce both molecular and tissue alterations, initiating multiple physiological processes which act simultaneously to stimulate cardiac regeneration. However, given the limited amount of clinical trials and mechanistic data, further research is warranted to elucidate the effect in humans and the complete MoA underlying the functional improvement.

## Appendix A. PICO Criteria

1. Develop a question following the PICO criteria:
  - Purpose: To describe efficacy and mode of action (MoA) of mesenchymal stem cell (MSC) therapy in patients with non-ischemic dilated cardiomyopathy (NIDCM)
  - Condition or domain being studied: MSC efficacy and MoA in NIDCM
  - Participants/populations being studied:
    - Clinical trials, which have evaluated the efficacy of MSCs in adults ≥ 18 years diagnosed with NIDCM
    - Clinical trials that evaluate the efficacy of MSCs in both NIDCM and ischemic heart disease, if the data from NIDCM patients can be extracted separately
    - Studies of MSC efficacy and/or molecular/cellular effects performed in animals of NIDCM
  - Intervention(s), exposure(s): We will include clinical trials and animal models of NIDCM, in which the intervention is administration of MSCs, regardless of administration route (intracoronary infusion, intramyocardial injection, intravenous injection).
    - The MSC therapy may consist of MSCs of varying origin (bone-marrow, adipose tissue, wharton’s jelly, umbilical cord), transplant type (autologous, allogeneic, syngeneic, xenogeneic), status (cryopreserved or fresh), number of cells administered and number of cell administrations (single or repeated injections—if repeated, it must be stated)
  - Comparator(s)/control: The study will include trials that have compared:
    - MSC therapy, as defined above, versus placebo, sham intervention, or no intervention
    - Different cell types or administration routes against each other
    - Modified/preconditioned MSCs to normal MSCs, only if the data can be extracted separately for the normal MSC group
  - Outcome: As we aim to describe and summarize the currently known MSC MoA in NIDCM, all outcomes (physiological mechanisms) will be eligible for inclusion. This is done to map current knowledge and inform future research. Key mechanisms of interest:
    - Angiogenesis
    - Fibrosis
    - Immune response
    - Chemotaxis
    - Apoptosis
  - Study design
    - Clinical trials
    - Animal models of NIDCM
2. Inclusion and exclusion criteria are determined prior to the literature search
  - Inclusion:
    - Non-ischemic injury
      1. NIDCM induced by autoimmune myocarditis
      2. Anthracycline-induced NIDCM
      3. Isoproterenol-induced non ischemic heart failure
      4. Genetic NIDCM
    - MSCs including BM-MSCs, hUCB-MSCs, AT-MSCs
    - Treatment consisting of exosomes, conditioned medium etc. from MSCs
  - Exclusion
    - Ischemic injury
    - Any other kind of stem cell therapy than MSCs, also iPS-MSCs
    - Modified MSCs (preconditioning, gene modified, differentiated etc.), unless data from a control group with “normal” MSCs can be extracted separately
    - Any kind of co-intervention, regardless of character
    - The following animal models; arrythmogenic and hypertrophic cardiomyopathies, TAC-model (HFpEF), pressure overload
    - Patients with arrythmogenic and hypertrophic cardiomyopathies
    - Reviews
    - Editorial comments

## Appendix B. PRISMA Checklist

**Section/Topic**	**#**	**Checklist Item**	**Reported on Page #**
TITLE			
Title	1	Identify the report as a systematic review, meta-analysis, or both.	1
ABSTRACT			
Structured summary	2	Provide a structured summary including, as applicable: background; objectives; data sources; study eligibility criteria, participants, and interventions; study appraisal and synthesis methods; results; limitations; conclusions and implications of key findings; systematic review registration number.	1
INTRODUCTION			
Rationale	3	Describe the rationale for the review in the context of what is already known.	1–2
Objectives	4	Provide an explicit statement of questions being addressed with reference to participants, interventions, comparisons, outcomes, and study design (PICOS).	Appendix A
METHODS			
Protocol and registration	5	Indicate if a review protocol exists, if and where it can be accessed (e.g., Web address), and, if available, provide registration information including registration number.	N/A
Eligibility criteria	6	Specify study characteristics (e.g., PICOS, length of follow-up) and report characteristics (e.g., years considered, language, publication status) used as criteria for eligibility, giving rationale.	2–3
Information sources	7	Describe all information sources (e.g., databases with dates of coverage, contact with study authors to identify additional studies) in the search and date last searched.	2–3
Search	8	Present full electronic search strategy for at least one database, including any limits used, such that it could be repeated.	2 + Appendix C
Study selection	9	State the process for selecting studies (i.e., screening, eligibility, included in systematic review, and, if applicable, included in the meta-analysis).	2–3
Data collection process	10	Describe method of data extraction from reports (e.g., piloted forms, independently, in duplicate) and any processes for obtaining and confirming data from investigators.	2–3
Data items	11	List and define all variables for which data were sought (e.g., PICOS, funding sources) and any assumptions and simplifications made.	3
Risk of bias in individual studies	12	Describe methods used for assessing risk of bias of individual studies (including specification of whether this was done at the study or outcome level), and how this information is to be used in any data synthesis.	3 + Appendix E
Summary measures	13	State the principal summary measures (e.g., risk ratio, difference in means).	N/A
Synthesis of results	14	Describe the methods of handling data and combining results of studies, if done, including measures of consistency (e.g., I^2^) for each meta-analysis.	N/A
Risk of bias across studies	15	Specify any assessment of risk of bias that may affect the cumulative evidence (e.g., publication bias, selective reporting within studies).	5
Additional analyses	16	Describe methods of additional analyses (e.g., sensitivity or subgroup analyses, meta-regression), if done, indicating which were pre-specified.	N/A
RESULTS			
Study selection	17	Give numbers of studies screened, assessed for eligibility, and included in the review, with reasons for exclusions at each stage, ideally with a flow diagram.	4
Study characteristics	18	For each study, present characteristics for which data were extracted (e.g., study size, PICOS, follow-up period) and provide the citations.	Appendix D
Risk of bias within studies	19	Present data on risk of bias of each study and, if available, any outcome level assessment (see item 12).	Appendix E
Results of individual studies	20	For all outcomes considered (benefits or harms), present, for each study: (a) simple summary data for each intervention group (b) effect estimates and confidence intervals, ideally with a forest plot.	N/A
Synthesis of results	21	Present results of each meta-analysis done, including confidence intervals and measures of consistency.	N/A
Risk of bias across studies	22	Present results of any assessment of risk of bias across studies (see Item 15).	Appendix E
Additional analysis	23	Give results of additional analyses, if done (e.g., sensitivity or subgroup analyses, meta-regression (see Item 16)).	N/A
DISCUSSION			
Summary of evidence	24	Summarize the main findings including the strength of evidence for each main outcome; consider their relevance to key groups (e.g., healthcare providers, users, and policy makers).	4–12
Limitations	25	Discuss limitations at study and outcome level (e.g., risk of bias), and at review-level (e.g., incomplete retrieval of identified research, reporting bias).	13
Conclusions	26	Provide a general interpretation of the results in the context of other evidence, and implications for future research.	13
FUNDING			
Funding	27	Describe sources of funding for the systematic review and other support (e.g., supply of data); role of funders for the systematic review.	13

## Appendix C. Literature Search

PubMed MeSH terms and free text terms used for the systematic literature search. The included terms presented in the table below are written and spelled as indexed in PubMed, thus providing the exact search strategy used in this review.

**MeSH**	**#1**	**AND**	**#4**
OR	Mesenchymal Stem Cells [MeSH]		Cardiomyopathy, Dilated [MeSH]
TX/ALL	#2		#5
OR	Mesenchymal Stem Cell * Bone Marrow Mesenchymal Stem Cells Bone Marrow Stromal Cell * Multipotent Bone Marrow Stromal Cells Adipose-Derived Mesenchymal * Adipose Derived Mesenchymal * Adipose Tissue-Derived Mesenchymal * Adipose Tissue Derived Mesenchymal * Mesenchymal Stromal Cell * Multipotent Mesenchymal Stromal Cells Mesenchymal Progenitor Cell * Wharton Jelly Cell * Wharton’s Jelly Cell * Bone Marrow Stromal Stem Cells		Dilated Cardiomyopath * Familial Idiopathic Cardiomyopath * Congestive Cardiomyopath * Idiopathic Dilated Cardiomyopath * Nonischemic dilated cardiomyopathy Doxorubicin-Induced Cardi * Doxorubicin-Induced Heart * Anthracycline-induced * Anthracycline cardio * Doxorubicin cardio * Doxorubicin heart *
OR			Heart Failure [MeSH]
			Cardiac Failure Heart Decompensation Right-Sided Heart Failure Right Sided Heart Failure Myocardial Failure Congestive Heart Failure Left-Sided Heart Failure Left Sided Heart Failure Non-ischemic heart failure Non Ischemic Heart Failure

Embase Medical subject headings and free text terms used for the systematic literature search.

**Subject Headings**	**#1**	**AND**	**#4**
OR	Mesenchymal Stem Cell		Congestive Cardiomyopathy
TX/ALL	#2		#5
OR	Mesenchymal Progenitor Cell Mesenchymal Stem Cells Stem Cell, Mesenchymal		Cardiomyopathy, congestive Cardiomyopathy, dilated Congestive heart disease Congestive myocardiopathy Congestive myocardiopathy, idiopathic Dilated cardiomyopathy Idiopathic congestive cardiomyopathy Idiopathic congestive myocardiopathy Idiopathic dilated cardiomyopathy Myocardiopathy, congestive Myocardiopathy, idiopathic congestive Doxorubicin-Induced Cardiomyopathy Doxorubicin-Induced Heart Failure Anthracycline-induced Cardiomyopathy Anthracycline cardiomyopathy Doxorubicin cardiotoxicity Doxorubicin heart
OR			Heart Failure
			Backward failure, heart Cardiac backward failure Cardiac decompensation Cardiac failure Cardiac incompetence Cardiac insufficiency Cardiac stand still Cardial decompensation Cardial insufficiency Chronic heart failure Chronic heart insufficiency Decompensatio cordis Decompensation, heart Heart backward failure Heart decompensation Heart incompetence Heart insufficiency Insufficientia cardis Myocardial failure Myocardial insufficiency

## Appendix D. Included Studies

**Table:**

**Study**	**Trial Type**	**NIDCM Phenotype**	**MSC Type and Concen-Tration**	**Admini-Stration Route**	**Functional Outcome**	**Molecular Outcome**
Carmona, 2017	Preclinical (Wistar rats, 8 weeks)	NIDCM induced by autoimmune myocarditis	Syngeneic BM-MNCs, BM-MSCs or AT-MSCs, 5 × 10^6^	Intramyocardial	BM-MSCs and AT-MSCs increased LVEF to 79.8% and 75.1%, respectively	BM-MSCs and AT-MSCs increased serum VEGF at 24 h (peak) and at four weeks post treatment. BM-MSC decreased BNP after four weeks and improved cardiac fiber organization, number of vessels and reduced fibrosis (IHC)
Deng, 2017	Preclinical (Sprague Dawley rats, 8 weeks)	Adriamycin induced NIDCM	Syngeneic BM-MSCs, 1 × 10^7^	Intravenous	BM-MSCs increased LVEF	BM-MSCs upregulated Cx43, MEF2 and GATA4 and downregulated, TGF-β and Col-I (qPCR). BM-MSCs reduced CVF (IHC)
Fatkhutdinov, 2009	Clinical (27 NIDCM patients)	Idiopathic NIDCM with LVEF ≤35%	Allogeneic BM-MSCs, dose N/A	Intracoronary	BM-MSCs increased walk distance and reduced NYHA class at one and three months follow-up	BM-MSCs reduced serum BNP one week after transplantation
Florea, 2020 (POSIEDON-DCM)	Clinical (34 NIDCM patients)	NIDCM with LVEF <40%	Allogeneic and autologous BM-MSCs, 1 × 10^8^	Trans-endocardial	LVEF was significantly increased in both males and females	BM-MSCs decreased serum TNF-α after six months in both males and females. EPC-CFU increased and FMD improved 3 months post treatment
Guo, 2013	Preclinical (C57/BL6 mice, 10 weeks)	Doxorubicin induced NIDCM	Syngeneic BM-MSCs, 5 × 10^7^	Intravenous	BM-MSCs increased FS% and reduced LVDd and LVEDP	BM-MSCs reduced cardiac fibrosis and CVF (IHC)
Hare, 2017 (Poseidon-DCM)	Clinical (37 NIDCM patients)	NIDCM with LVEF <40%	Allogeneic and autologous BM-MSCs, 1 × 10^8^	Trans-endocardial	Allogeneic BM-MSCs increased LVEF to a greater degree than autologous BM-MSCs	Serum TNF-α decreased to a greater extent in the allogeneic group compared to the autologous group at six months follow-up. EPC-CFU increased in the allogeneic group compared to the autologous at three months follow-up. Both groups had a reduced percentage of T and B cell subtypes, normally associated with chronic inflammation, at six months follow-up
Kong, 2010	Preclinical (Wistar rats, 3 months, 250 g)	Adriamycin induced NIDCM	Transplant type N/A, BM-MSCs, 2 × 10^6^	Intravenous for three days	BM-MSCs increased LVEF	BM-MSCs significantly reduced cardiac norepinephrine content and increased GAP-43, ChaT, and SYN density (IHC and WB)
Li, 2009	Preclinical (Wistar rats, 180–200 g)	Isoproterenol induced HF	Syngeneic BM-MSCs, 3 × 10^6^	Intramyocardial	BM-MSCs increased LVEF and FS	BM-MSC attenuated myocardial fibrosis (IHC) and upregulated adrenomodullin (qPCR)
Mao, 2017	Preclinical (Sprague Dawley rats, 180 g)	Doxorubicin induced NIDCM	Xenogeneic hUCB-MSCs or CM, 2.5 × 10^5^ (low dose) or 1 × 10^6^ (high dose) or 2.0 mL (CM)	Intramyocardial	Both low-dose and high-dose of hUCB-MSCs increased FS% and LVEF.	hUCB-MSCs attenuated mitochondrial swelling and maintained sarcolemma integrity (IHC). BM-MSCs increased serum LIF at both doses, HGF, GM-CSF, and VEGF at low dose and reduced BNP, cTNI (ELISA). Treatment increased HGF, VEGF, IGF-1 (qPCR)
Mörschbächer, 2016	Preclinical (New Zealand rabbits, 3–4 months, 2–3.5 kg)	Doxorubicin induced NIDCM	Syngeneic AT-MSCs, 1 × 10^6^	Intramyocardial	No significant change in LVEF	AT-MSCs reduced histological lesions (IHC)
Premer, 2019 (POSEIDON-DCM)	Clinical (21 NIDCM patients)	NIDCM with LVEF <40%	Allogeneic and autologous BM-MSCs, 1 × 10^8^	Trans-endocardial	N/A	Allogeneic BM-MSCs increased EPC-CFUs and decreased plasma SDF-1α in both treatment groups. Plasma TNF-α negatively correlated with EPC-CFUs
Premer, 2015 (POSEIDON-DCM + TRIDENT)	Clinical (12 NIDCM patients)	Idiopathic NIDCM (inclusion criteria from POSEIDON-DCM)	Allogeneic and autologous BM-MSCs, 1 × 10^8^	Trans-endocardial	N/A	Allogeneic BM-MSCs led to increased EPC-CFUs and improved FMD compared to autologous BM-MSCs
Rieger, 2019 (POSEIDON-DCM)	Clinical (34 NIDCM patients)	NIDCM with LVEF <40%	Allogeneic and autologous BM-MSCs, 1 × 10^8^	Trans-endocardial	BM-MSCs increased LVEF in patients negative for any pathological variants (V-) and variants of uncertain significance at one year follow up, and improved MLHFQ score and NYHA class in V- patients only	N/A
Shabbir, 2009	Preclinical (TO2 (cardiomyopathic) male hamsters, 4 months)	TO2 (cardiomyopathic) male hamsters	Xenogeneic BM-MSCs, 0.25 × 10^6^, 1 × 10^6^ or 4 × 10^6^ or 0.8 mL CM	Two intramuscular injections (hamstring muscle) with 2 weeks interval or CM three times per week for 4 weeks	BM-MSCs increased FS% at all concentrations. 4 × 10^6^ BM-MSCs increased FS% to the greatest degree	BM-MSCs decreased myocyte diameter, apoptotic myocytes, fibrosis (IHC), and circulating cTnI (ELISA). BM-MSCs downregulated Col3a1, MMP-9, MMP-13, TIMP-2, TIMP-3 (qPCR)
Xiao, 2017	Clinical (53 NIDCM patients)	NIDCM with LVEF <40%	Autologous BM-MSCs or BM-MNCs, number of adherent cells in passage 3	Intramyocardial	LVEF, LVEDd, NYHA class were improved after three and 12 months in both groups. Myocardial perfusion had increased in the BM-MSC group	N/A
Yu, 2014	Preclinical (Sprague Dawley rats, 8 weeks)	Doxorubicin induced NIDCM	Syngeneic BM-MSCs, 5 × 10^6^	Intravenous every other day for 10 days	Repeated infusions of BM-MSCs increased LVEF to 79.6% and decreased LVEDd	BM-MSCs reduced CVF and Col-I/III ratio (IHC) MSCs downregulated Col-I, AT1, CYP11B2, TGF-β1 and upregulated Col-III (qPCR)
Yu, 2015	Preclinical (Sprague Dawley rats, 37 g)	Furazolidone induced NIDCM	Syngeneic BM-MSCs, 1 × 10^5^	Intramyocardial	BM-MSCs increased LVEF to 74%	BM-MSCs reduced CVF (IHC), Col-I/III ratio and downregulated myocardial TGF-β1 (qPCR)
Zhang, 2019	Preclinical (Lewis rats)	NIDCM induced by autoimmune myocarditis	Xenogeneic hUCB-MSCs, 1 × 10^6^	Intravenous	N/A	hUCB-MSCs decreased myocardial fibrosis (IHC) and activity of TGF-β1/ERK1/2 signaling
Labelled cells						
Abd Allah, 2017	Preclinical (Male albino rats, 150–200 g)	Doxorubicin induced NIDCM	Xenogeneic, PKH26-labelled c hUCB-MSCs, 1 × 10^6^	Intravenous	EDP, dP/dt max, and dP/dt min increased after six weeks	hUCB-MSCs decreased serum cTnI (ELISA) and collagen area (IHC)
Abdelmonem, 2019	Preclinical (Wistar rats, 12 weeks old)	Isoprenaline induced HF	Syngeneic, PKH26-labelled BM-MSCs, 1 × 10^7^	Intravenous	BM-MSC increased LVEF to 74.47% and decreased LVESd	BM-MSCs decreased fibrosis and increased GATA4, desmin and cTNI (IHC) and increased eNOS (WB) and MEF2c (qPCR)
Ammar, 2015	Preclinical (Wistar rats, 200–220 g)	Diabetic mellitus + doxorubicin induced NIDCM	Xenogeneic GFP labelled hBM-MSCs, 2 × 10^6^, or GFP labelled hAT-MSCs, 1 × 10^6^	Intravenous	Both MSC types increased FS% and decreased arterial blood pressure (systolic and diastolic)	Both MSC groups led to an increased number of capillaries and decreased immune cell infiltration, collagen deposition and αSMA (IHC)
Chen, 2010	Preclinical (Inbred Japanese rabbits, 1800–2000 g)	Doxorubicin induced NIDCM	Autologous BrdU labelled BM-MSCs, 5 × 10^5^	Intramyocardial	BM-MSCs increased LVEF to 68.38% and decreased LVESd	BM-MSCs were present in the myocardium four weeks after treatment, indicated by BrdU (IHC)
Gong, 2016	Preclinical (cTnTR141W transgenic mice, 4 months)	Genetic NIDCM	Xenogeneic eGFP labelled hUCB-MSCs, 1.5 × 10^6^	Intramyocardial	hUCB-MSCs increased LVEF to 56.96% and decreased heart weight/body weight, LVEDd, LVESd	hUCB-MSCs reduced CVF, cytoplasmic vacuolisation and apoptotic nuclei and increased CD31^+^ vessels and αSMA^+^ aterioles (IHC). hUCB-MSCs increased Bcl-2/Bax ratio (WB), IGF-1 and VEGF and reduced serum CRP (ELISA)
Li, 2018	Preclinical (Male Wistar rats, 180–200 g)	Isoproterenol induced HF	Syngeneic eGFP + DAPI labelled BM-MSCs, 5 × 10^6^	Intramyocardial	BM-MSCs increased LVEF to 69.24% and reduced LVESd	BM-MSCs decreased CVF (IHC), upregulated HGF and downregulated Col-I and III, MMP-2, MMP-9, TNF-β (qPCR), MMP-2 and MMP-9 (WB). BM-MSCs were present in the myocardium after four weeks
Li, 2008	Preclinical (Male Wistar rats, 180–200 g)	Isoproterenol induced HF	Syngeneic DAPI labelled BM-MSCs, 3 × 10^6^	Intramyocardial	BM-MSCs increased LVEF to 78.51% and decreased LVESd	BM-MSCs increased HGF (qPCR and WB) and decreased CVF (IHC), Col-I and III, MMP-2 and MMP-9 (qPCR), Pro MMP-2, Active MMP-2 and MMP-9 (WB)
Mohamed, 2015	Preclinical (Male Wistar rats, 170–190 g)	Isoproterenol induced HF	Syngeneic PKH26 labelled BM-MSCs, 1 × 10^6^	Intravenous	BM-MSCs increased LVEF to 58.33%	BM-MSCs reduced cardiac fibrosis (IHC) and increased eNOS, Cx43 (WB). BM-MSCs reduced caspase 3 (WB) and TGF- β (ELISA)
Nagaya, 2006	Preclinical (Male Lewis rats, 220–250 g)	NIDCM induced by autoimmune myocarditis	Syngeneic BM-MSCs, 5 × 5^6^	Intramyocardial	BM-MSCs increased FS% and decreased LVEDP, LVDd	BM-MSCs increased myocardial capillary density and decreased CVF (IHC). BM-MSCs reduced MMP-2 (WB)
Psaltis, 2010	Preclinical (Merino wether sheeps, 50 kg)	Doxorubicin induced NIDCM	Allogeneic GFP labelled Mesenchymal progenitor cells (MPCs), 1 × 10^9^ ± 5 × 10^6^	Trans-endocardial	MPCs increased LVEF to 39.2%	MPC treatment reduced CVF and increased the density of karyokinetic cardiomyocytes and myocardial arterioles (IHC)
Yang, 2013	Preclinical (Female Wistar rats, 210–240 g)	Adriamycin induced NIDCM	Syngeneic BrdU labelled BM-MSCs, 5 × 10^6^	One or two intravenous injections (1-day interval	Only two doses significantly increased LVEF. Two doses of BM-MSCs increased LVEF to 75.4%. BM-MSCs reduced mortality, LVESd and LVEDd, which were significantly improved by double infusion	BM-MSCs led to upregulation of VEGF (qPCR). Two injections decreased CVF (IHC) and serum BNP (ELISA). BM-MSCs improved fiber alignment (IHC) All parameters significantly improved by double infusion BrdU labelled BM-MSCs were present in the myocardium after Four weeks
Zhang, 2013	Preclinical (Wistar rats, 6–7 weeks, 200–220 g)	Adriamycin induced NIDCM	Syngeneic BrdU labelled BM-MSCs, 1 × 10^7^	Intravenous	BM-MSCs increased LVEF ti 55.56% and decreased LVESd	BM-MSCs increased GATA-4, cTnI, Cx43 (IHC) and decreased serum BNP (ELISA)
Zhou, 2007	Preclinical (New Zealand rabbits, 1.9 kg)	Adriamycin induced NIDCM	Autologous DAPI labelled BM-MSCs, 4 × 10^6^	Intramyocardial	No significant improvement in heart function.	BM-MSCs were present in the myocardium after two weeks and increased Bcl-2 (IHC)

Appendix D Included studies. Abbreviations ordered according to the table; Non-ischemic dilated cardiomyopathy (NIDCM), Bone marrow mononuclear cells (BM-MNCs), Bone marrow derived mesenchymal stem cells (BM-MNCs), Adipose tissue–derived mesenchymal stem cells (AT-MSCs), Left ventricular ejection fraction (LVEF), Vascular endothelial growth factor (VEGF), Immunohistochemistry (IHC), Connexin43 (Cx43), Myocyte enhancer factor-2 (MEF2), Transcription factor GATA-4 (GATA4), Transforming growth factor beta (TGF-β), Collagen (Col), Quantitative Polymerase Chain Reaction (qPCR), Collagen volume fraction (CVF), New York heart association (NYHA), Brain natriuretic peptide (BNP), Tumor necrosis factor α (TNF-α), Percutaneous Stem Cell Injection Delivery Effects on Neomyogenesis in Dilated Cardiomyopathy (POSEIDON-DCM), Endothelial progenitor cell-colony forming units (EPC-CFU), Flow mediated vasodilation (FMD), Fractional shortening (FS%), Left ventricular diastolic dimension (LVDd), Left ventricular end-diastolic pressure (LVEDP), Growth Associated Protein 43 (GAP-43), Cholineacetyltransferase (ChaT), Synaptophysin (SYN), Western blotting (WB), Heart failure (HF), Human umbilical cord blood MSCs (hUCB-MSCs), Conditioned medium (CM), Leukocyte inhibitory factor (LIF), Hepatocyte growth factor (HGF), Granulocyte- macrophage colony-stimulating factor (GM-CSF), Insulin-like growth factor-1 (IGF-1), Troponin T (cTNI), Enzyme-linked immunosorbent assay (ELISA), Stromal cell-derived factor 1 alpha (SDF-1α), The Transendocardial Stem Cell Injection Delivery Effects on Neomyogenesis Study (TRIDENT), The Minnesota living with heart failure questionnaire (MLHFQ), Collagen Type III Alpha 1 Chain (Col3a1), Matrix metalloproteinase (MMP), Tissue inhibitor of metalloproteinase (TIMP), Left ventricular end-diastolic diameter (LVEDd), Angiotensin II receptor type 1 (AT1), Cytochrome P450 Family 11 Subfamily B Member 2 (CYP11B2), Extracellular signal-regulated kinase (ERK), End diastolic pressure (EDP), Left ventricle maximal pressure rise in early systole (dP/dt max), Left ventricle maximal decline of pressure in early diastole (dP/dt min), Left ventricular end-systolic diameter (LVESd), Bromodeoxyuridine (Brdu), Endothelial NOS (eNOS), Green fluorescent protein (GFP), Alpha smooth muscle actin (αSMA), B-cell lymphoma 2 (Bcl-2), Bcl-2 Associated X, Apoptosis Regulator (Bax), C-reactive protein (CRP), Tumor necrosis factor β (TNF-β), 4′,6-diamidino-2-phenylindole (DAPI), Red fluorescent cell linker (PKH26).

## Appendix E. Risk of Bias Assessment

Appendix E Risk of Bias assessment. Green (+) indicates low risk of bias, red (-) high risk of bias and yellow (?) not reported.
